# Synthesis, DNA-Binding, Anticancer Evaluation, and Molecular Docking Studies of Bishomoleptic and Trisheteroleptic Ru-Diimine Complexes Bearing 2-(2-Pyridyl)-quinoxaline

**DOI:** 10.1155/2021/5599773

**Published:** 2021-05-12

**Authors:** Sofia Balou, Athanasios Zarkadoulas, Maria Koukouvitaki, Luciano Marchiò, Eleni K. Efthimiadou, Christiana A. Mitsopoulou

**Affiliations:** ^1^Inorganic Chemistry Laboratory, Chemistry Department, National and Kapodistrian University of Athens, Panepistimiopolis, Zografou 157 71, Greece; ^2^Dipartimento di Scienze Chimiche, della Vita e della Sostenibilità Ambientale, Università degli Studi Parma, Parco Area delle Scienze 17A, I43124 Parma, Italy

## Abstract

Herein, we report the synthesis and characterization of a bishomoleptic and a trisheteroleptic ruthenium (II) polypyridyl complex, namely, [Ru(bpy)2(2, 2′-pq)](PF6)2 (1) and [Ru(bpy) (phen) (2, 2′-pq)](PF6)2 (2), respectively, where bpy = 2,2′-bipyridine, phen = 1,10-phenanthroline, and 2, 2′-pq = 2-(2′-pyridyl)-quinoxaline. The complexes were characterized by elemental analysis, TGA, ^1^H-NMR, FT-IR, UV-Vis, emission spectroscopy, and electrochemistry. Their structures were confirmed by single-crystal X-ray diffraction analysis. Complexes 1 and 2 were crystalized in orthorhombic, Pbca, and monoclinic, P21/*n* systems, respectively. Various spectroscopic techniques were employed to investigate the interaction of both complexes with calf thymus DNA (CT-DNA). The experimental data were confirmed by molecular docking studies, employing two different DNA sequences. Both complexes, 1 and 2, bind with DNA via a minor groove mode of binding. MTT experiments revealed that both complexes induce apoptosis of MCF-7 (breast cancer) cells in low concentrations. Confocal microscopy indicated that 2 localizes in the nucleus and internalizes more efficiently in MCF-7 than in HEK-293.

## 1. Introduction

Ruthenium diimine complexes have long attracted interest due to their various applications in catalysis [1, 2] and in metal-based drugs [3]. More specifically, ruthenium diimine complexes exhibit telomerase and topoisomerase inhibition [4, 5] as well as protease activity [6]. They can induce apoptosis in cancer cells in various stages of the replication cell cycle via intercalation or after photoexcitation, leading to applications in photodynamic therapy [7, 8].

Focusing on the ligand framework, peripheral functionalization of diimines has led to the development of multipotent metal-based drugs containing second and third row transition metals bearing dipyridophenazine (dppz) [9], 1,10-phenanthroline-5,6-dione [10, 11], or quinoxaline ligands [12–14]. More specifically, 2, 2′-pq proved a versatile ligand that can bind to a plethora of transition metals [15]. The quinoxaline moiety is found in many molecules of medicinal interest that exhibit antibacterial, antiviral, antifungal, anthelmintic, and anticancer properties [16, 17].

When coordinated to metal ions, the resulting complexes have interesting bioinorganic applications. For example, [ReOCl_3_(2, 2′-pq)] can efficiently cleave plasmid pBR 322DNA upon irradiation by generating singlet oxygen and hydroxyl radicals [18]. Furthermore, incorporation of 2, 2′-pq in Pt-diamine complexes [19] and study against murine leukemia results in IC_50_ values in the range of ∼40 *μ*M. Square planar and octahedral Rh(I) and Rh(II) complexes comprise 2, 2′-pq as a ligand exhibit activity against the platelet-activating factor (PAF) [20], while organometallic Ru(II) complexes with arene ligands and 2, 2′-pq [21] bind to oligonucleotides and exhibit cytotoxic properties with IC_50_ < 1 *μ*M. Ruthenium complexes bearing 2, 2′-pq ligand have also been reported [22], with both Δ- and Λ-enantiomers of [Ru(bpy)_2_(2, 2′-pq)]^2+^ binding to oligonucleotide duplex d(CGCGAATTCGCG)_2_ [23].

Trisheteroleptic diimine Ru complexes for bioinorganic chemistry applications are quite scarce in the literature, one example being the [Ru(bpy) (dppn) (CH_3_CN)_2_]^2+^ reported by Albani et al. [24], which exhibits a dual action, both ^1^O_2_ generation and ligand exchange after irradiation. In another instance, a library of 28 Ru(II) trisheteroleptic diimine complexes was prepared using solid-phase synthesis to yield [Ru(phen-Me_2_) (dppz-Me_2_) (phen-CONH_2_)]^2+^ as a 500-time more potent inhibitor against acetylcholinesterase than homoleptic [Ru(phen)_3_]Cl_2_ [25]. Moreover, Bhat et al. [26] recently reported trisheteroleptic Ru-complexes that exhibit the “molecular light switch” effect and are toxic against HeLa and HL-60 cell lines, while cellular uptake is localized in the cell nucleus. These reports show the high potential of Ru-trisheteroleptic complexes in the design of multimodal metallotherapeutic agents [27].

**Scheme 1:**
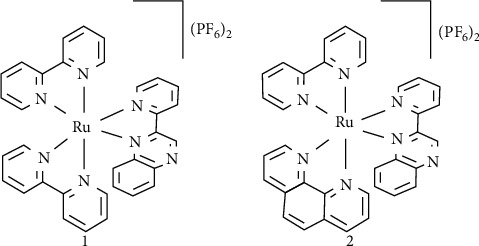
Chemical structures of complexes under study in this work (complexes 1 and 2).

Inspired by the proven affinity of 2, 2′-pq-containing metal complexes for DNA and the diversity of opportunities opening from incorporating a trisheteroleptic ligand manifold around Ru(II), in this study, we report the preparation and characterization of the trisheteroleptic [Ru(bpy) (phen) (2, 2′-pq)](PF_6_)_2_ complex along with its [Ru(bpy)_2_(2, 2′-pq)](PF_6_)_2_ counterpart. The latter was studied because although its fully structural characterization has not to be presented before, its binding mode to DNA has been reported; thus, it can be a good comparison for our results. As a consequence, both complexes (Scheme 1) have been structurally characterized, and the interaction of the trisheteroleptic complex with CT-DNA is reported. Moreover, both complexes were tested against MCF-7 and HEK-293 cancer cells, while molecular docking analysis has been performed to obtain detailed information on the binding mode of complexes 1 and 2 with CT-DNA.

## 2. Experimental

### 2.1. Materials and Methods

#### 2.1.1. General Considerations

Solvents and reagents were used as received from Aldrich, Fisher, and Merck. CDCl_3_ was further purified by distillation over K_2_CO_3_ and was stored over 4A molecular sieves in dark. Elemental analyses were obtained by a LECO-183 CHNS analyzer. IR spectra were conducted on a 2.0 cm^−1^ resolution Shimadzu IR Affinity-1 spectrometer using KBr pellets. Varian Unity Plus instrument (300 MHz) was used for obtaining NMR spectra, the correction of which was carried out by implementing the solvent peak as an internal standard. Both Hitachi U-2000 and Varian Cary 3E spectrophotometers were used for recording the UV-Vis spectra. The path length of the used quartz cuvettes was 1.0 cm. 2-2′-pyridyl-quinoxaline (2, 2′-pq) [28], cis-[Ru(bpy)_2_Cl_2_] [29], and cis-[Ru(DMSO)_4_Cl_2_] [30] were prepared as mentioned in the literature, and all analytical data agree with those reported. HRMS spectra were recorded using a Q-TOF mass spectrometer (Maxis Impact, Bruker Daltonics, Bremen, Germany). TGA was performed with a TGA/DCS1 Mettler Toledo analyzer. More details on experimental procedures can be found in the supporting information.

#### 2.1.2. Electrochemistry

All electrochemical experiments were conducted on AFCBP1 Pine Instrument Company potentiostat. A three-electrode cell was used for obtaining cyclic voltammograms. The used electrodes were a glassy carbon working one, a platinum wire counter electrode, and a reference electrode, namely, Ag/AgCl (KCl 3M). The latter was replaced in some measurements by an Ag wire pseudoreference electrode. Before each measurement, the working electrode was refined with alumina paste (1 *μ*m) on a polishing cloth. Before each measurement, the solution was purged with argon gas for 10 min, and ferrocene was used as an internal standard. All voltammograms were recorded at a 100 mV/s scan rate unless otherwise noted.

#### 2.1.3. X-ray Structure Determination

Table 1 summarizes the data collection and structure refinement for 1 and 2. X-ray structure determination was performed on suitable single red crystals of 1 and 2 which were grown by layering their concentrated dichloromethane solution with n-hexane. Single crystal data were collected with a Bruker Smart Breeze area detector diffractometer (Mo Ka radiation, *λ* = 0.71073 Å). The intensity data were integrated from several series of exposure frames (0.3 width) covering the sphere of reciprocal space [31]. The structure was solved SHELXT [32] and refined on F2 with full-matrix least squares (SHELXL-2014) [33], using Olex2 software package [34]. Graphical material was prepared with the Mercury 3.10 program [35]. CCDC deposition number is 2052212-2052213.

### 2.2. Synthesis of Ru Complexes

#### 2.2.1. Synthesis of [Ru(bpy)_2_(2, 2'-pq)](PF_6_)_2_ (1)

100 mg (0.206 mmol) of cis-[Ru(bpy)_2_Cl_2_] and 51.2 mg (1.2 eq.) of 2, 2′-pq were dissolved in an 8 : 2 ethanol-water 20 mL mixture, and the solution was refluxed overnight. The solvents were removed in a rotary evaporator, and a saturated solution of KPF_6_ in water was added (15 mL), which resulted in the formation of a red precipitate. The mixture was filtered in a sintered funnel, washed with water and ether, and air-dried. The resulting solid was dissolved in a minimum amount of MeCN and subjected to column chromatography (neutral alumina, MeCN solvent). The orange band eluting first was isolated, concentrated, and addition of ether afforded 160 mg of a red solid. Yield: 85%. Anal. found (calc.) for C_33_H_25_F_12_N_7_P_2_Ru: C, 43.10 (43.53); H, 2.81 (2.77); and N, 10.80 (10.77). ^1^H-NMR (acetone-d_6_, ppm): 10.10 (s, 1H, 2, 2′-pq), 9.26 (d, 1H, 2, 2′-pq), 8.90 (d, 2H), 8.76 (d, 1H, 2, 2′-pq), 8.64 (d, 1H, 2, 2′-pq), 8.34–8.09 (m, 9H), 7.96 (d, 1H), 7.89 (m, 1H), 7.79 (d, 1H), 7.66 (m, 2H), and 7.57–7.47 (m, 5H). IR (KBr, cm^−1^): 842 (PF_6_^–^) and 761 (PF_6_^–^). HRMS-ESI, positive in MeOH, and m/z: 310.5596/2 (calc., 310.56).

#### 2.2.2. Synthesis of [Ru(bpy) (phen) (2, 2'-pq)](PF_6_)_2_ (2)

33 mg (0.0649 mmol) cis-[Ru(bpy) (phen)Cl_2_] (details in SI ) and 16.14 mg (1.2 eq) of 2, 2′-pq were dissolved in an 8 : 2 10 mL ethanol-water mixture, and the solution was refluxed overnight. The solvents were removed in a rotary evaporator, and a saturated solution of KPF_6_ in water was added (15 mL), which resulted in the formation of a brown-orange precipitate. The mixture was filtered in a sintered funnel, washed with water and ether, and air-dried. The resulting solid was dissolved in a minimum amount of MeCN and subjected to column chromatography (neutral alumina, MeCN solvent). The orange band eluting first was isolated, concentrated, and addition of ether afforded 51 mg of a red-orange solid. Yield: 84%. Anal. found (calc.) for C_35_H_25_F_12_N_7_P_2_Ru: C, 44.64 (44.98); H, 2.81 (2.70); and N, 10.56 (10.49). ^1^H-NMR (DMSO-d_6_, ppm): 10.19 (s, 1H, 2, 2′-pq), 9.30 (d, 1H, 2, 2′-pq), 8.85–8.74 (m, 6H), 8.44 (t, 2H), 8.26 (m, 2H), 8.13–7.97 (m, 2H), 7.87–7.69 (m, 4H), 7.52–7.30 (m, 6H), and 7.16 (m, 1H). IR (KBr, cm^−1^): 840 (PF_6_^–^) and 770 (PF_6_^–^). HRMS-ESI, positive in MeOH, and m/z: 322.5589/2 (calc.: 322.561).

### 2.3. DNA-Binding Studies of Complex 2

All experiments involving DNA interactions with complex 2 were carried out in Tris-HCl buffer with pH = 7.0 which was prepared by dissolving 0.394 g (2.5 mmol) of Tris(hydroxymethyl)aminomethane hydrochloride (Tris-HCl) and 1.461 g (25 mmol) of NaCl in 500 mL of Milli-Q water. A detailed description of the methodology is given in SI under the DNA binding studies including circular dichroism (CD) measurements, absorption titration, viscosity measurements, and fluorescence emission spectroscopy.

#### 2.3.1. In Vitro Cytotoxicity Studies and Confocal Microscopy

The MTT assay was used for testing the growth inhibition of MCF-7 and HEK-293 cells for compounds 1 and 2 and cis-platin [36]. Confocal laser microscopy was used for studying the cellular uptake of each compound. A detailed description of the methods is also provided in SI.

#### 2.3.2. Molecular Docking Study

Docking studies were carried out using MGL tools 1.5.4 with AutoGrid4 and AutoDock4 to perform blind docking calculations between both ruthenium (II) complexes and DNA sequence. A full description is provided in SI together with the output files.

## 3. Results and Discussion

### 3.1. Synthesis

**Scheme 2:**
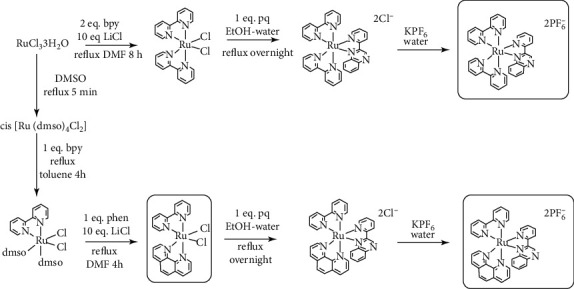
Preparation routes for complexes 1 and 2.

The preparation of desired complexes was achieved as shown in Scheme 2. To achieve the preparation of trisheteroleptic ruthenium complexes, several ways have been proposed in the literature [37–39], mainly by using carbonyl Ru complexes or photochemical processes. However, we found that the most convenient and reliable method to obtain the trisheteroleptic Ru complex was by stepwise substitution of labile ligands, with each intermediate easily isolable and characterized. Thus, initially, two DMSO molecules were substituted by one bpy molecule in the cis-[Ru(DMSO)_4_Cl_2_] precursor, as mentioned in the literature [40]. The use of CHCl_3_ resulted in low yields and insoluble products; therefore, refluxing toluene was chosen as a solvent, instead [41]. Reaction of the cis-[Ru(bpy) (DMSO)_2_Cl_2_] with one eq. phen in refluxing DMF in the presence of 10 eq. LiCl resulted in the formation of the heteroleptic cis-[Ru(bpy) (phen)Cl_2_] complex. In turn, this intermediate reacted with one eq. of 2,2′-pq ligand in refluxing EtOH-water mixture which, after chloride-PF_6_^−^ exchange, afforded the desired trisheteroleptic complex 2.

Both complexes 1 and 2 were thermally stable up to 300°C, as evidenced by their thermograms (see SI for detailed discussion, Figure S1 and Table S1), and their purity was identified by elemental analysis. The high-resolution mass spectra for both complexes, in the positive mode in methanol, and their corresponding isotope distribution patterns are in accordance with the proposed structure (Scheme 1).

### 3.2. Electronic Absorption and Emission Spectroscopy

UV-Vis spectra of complexes 1 and 2 measured in acetonitrile display typical MLCT transitions in the visible region of the spectrum. More specifically, both complexes display absorption maxima at 507 nm and 428 nm with molar absorptivity values of 5000–10000 M^−1^·cm^−1^, respectively, both assigned as transitions to singlet MLCT excited states (Ru^II^⟶*π*^*∗*^_diimine_) [42]. These bands are separated by 79 nm due to the different energy of diimine orbitals that leads to two different transitions. However, the difference in energy of the orbitals of the three distinct diimines in the trisheteroleptic complex 2 is not large enough to lead to the appearance of three bands [39]. The long tail after 500 nm in both spectra indicates a transition to the spin-forbidden ^3^MLCT state, as reported for similar complexes [43].

**Figure 1:**
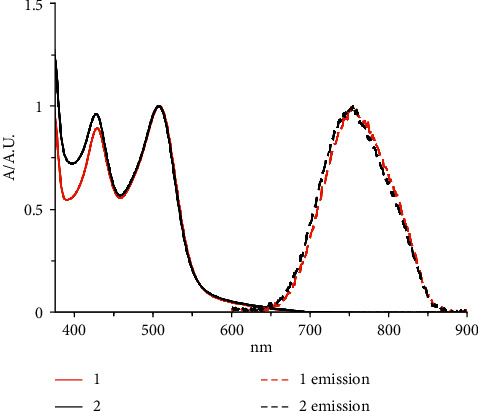
Normalized UV-Vis and emission spectra of complexes 1 (red trace) and 2 (blue trace) in acetonitrile.

Both complexes display a single broad emission band when excited at 500 nm in acetonitrile solution, with an emission wavelength of 750 nm. This emission arises from the lowest-lying ^3^MLCT state that is formed via intersystem crossing from a ^1^MLCT after absorption [42, 44]. Normalized absorption and emission spectra for complexes 1 and 2 are shown in Figure 1 with results summarized in Table 2.

### 3.3. Electrochemical Data

**Figure 2:**
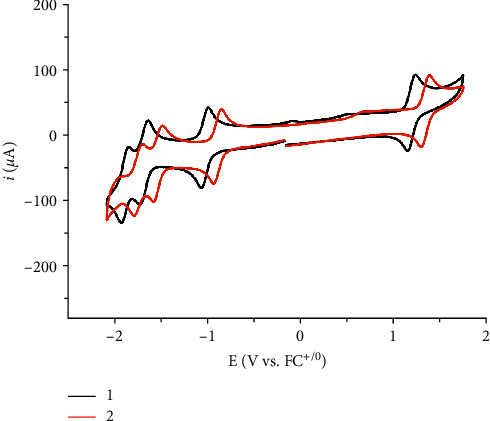
Cyclic voltammograms for complexes 1 (black trace) and 2 (red trace) in acetonitrile.

Cyclic voltammograms of 1 and 2 in MeCN are shown in Figure 2, and *E*_1/2_ values are given in Table 3. Both complexes display a set of three reversible reductions and one reversible oxidation. The reductions are ligand-based while the oxidation has been designated as Ru^II/III^ oxidation [42, 45].

(1) $$\begin{array}{c} {\left[\text{RuII}\left(\text{L}1\right)\left(\text{L}2\right)\left(\text{L}3\right)\right]}^{2+}{+\text{e}}^{-}⟶{\left[\text{RuII}\left({\text{L}1}^{\cdot -}\right)\left(\text{L}2\right)\left(\text{L}3\right)\right]}^{+}. \end{array}$$

Replacing a bpy ligand of 1 with a phen ligand yields complex 2, with the reduction potentials being slightly affected. More specifically, the first reduction for complex 1 is located at −1.023 V vs Fc^+/0^, while for complex 2, it occurs at −0.889 V vs. Fc^+/0^. This trend is continued for the next reductions, with the reductions of complex 2 occurring at ∼140 mV less-negative values. This can be explained in terms of the *π*^*∗*^-accepting ability of the diimines. Since phen has lower lying *π*^*∗*^-orbitals than bpy due to the increased delocalization over one more fused aromatic ring, complex 2 can be more easily reduced than 1. The reversible oxidation lies at 1.190 V and at 1.343 V vs Fc^+/0^ for 1 and 2, respectively. Increased back donation from Ru^II^ orbitals to the *π*^*∗*^ orbitals of diimine stabilizes the +2 oxidation state resulting in more positive potential required to oxidize the trisheteroleptic complex.

### 3.4. Crystal Structure Determination

**Figure 3:**
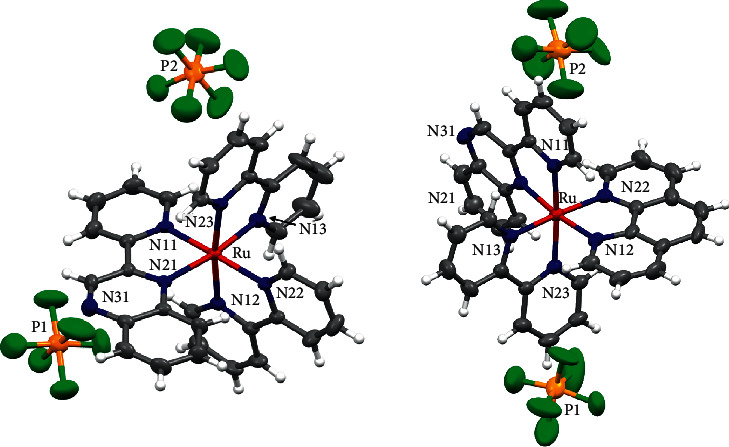
X-ray structures for complexes 1 and 2. Thermal ellipsoids are depicted at the 30% probability level. Solvent molecules of crystallization were omitted for clarity.

We isolated single crystals suitable for X-ray structure determination for complexes 1 and 2. The molecular structures are shown in Figure 3, and selected bond distances are summarized in Table 4. In both complexes, the metal adopts a distorted octahedral coordination environment, according to the presence of the three bidentate ligands forming five-membered chelate rings with bite angles of approximately 78°. Furthermore, in both complexes, the metal-N_quinoxaline_ bond length is elongated with respect to the metal-N_pyridyl_ bond length, and this difference can be explained in terms of the less *σ*-donating ability of the quinoxaline N-atom with respect to the N_pyridyl_ moiety. In support of this observation, it is instructive to compare the crystal structures of several late-transition metal complexes that contain the 2, 2′-pq ligand, which were reported by us, in particular, complexes with Mo [46], Re [18, 47, 48], and W [49, 50]. In all cases, the same trend for the metal-N_quinoxaline_ and metal-N_pyridyl_ bond lengths, found in 1 and 2, is also found for these complexes (Table 5). In addition and by inspecting in more detail the structural information between 1 and 2, a shorter metal-N_quinoxaline_ bond length in 2 (2.097 Å) when compared to 1 (2.119 Å) can be evidenced, pointing to a possible increase in the metal-N_quinoxaline_ bond strength in 2. This geometric observation is in line with electrochemical measurements discussed earlier, which would imply a more pronounced back-donation of the quinoxaline moiety in complex 2.

### 3.5. DNA-Binding Studies

#### 3.5.1. Circular Dichroism Experiments

**Figure 4:**
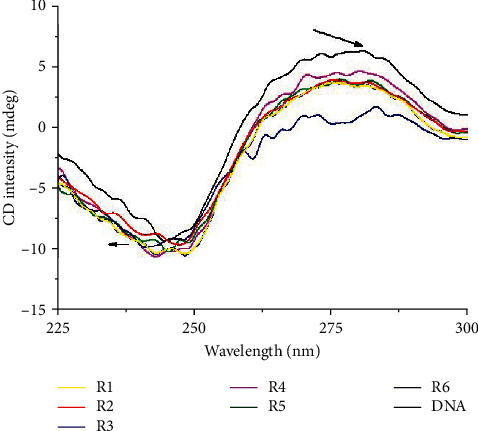
CD spectrum of DNA in the presence of complex 2.

Circular dichroism studies enabled the monitoring of the extent of conformation of DNA in the presence of increasing concentrations of complex 2, since it is well known that DNA CD spectrum alters in the presence of a binding molecule. This is either due to the pairing of DNA and ligand transitions or due to modifications in the DNA base coupling followed by changes in the geometry. As CD spectra reveal (Figure 4), addition of complex 2 changes the intensity of both spectrum bands of CT-DNA's B-form. In the presence of complex 2, the positive band at 280 nm decreases significantly in intensity, which suggests nonclassical intercalation [53]. Additionally, the negative peak at 246 nm increases, and a slight blue shift is concurrently noticed. Thus, the raise of negative band leads to gradual unfolding of DNA. Furthermore, the decrease observed at the maximum positive peak indicates a more compact structure [54, 55]. The consequent changes in the CD spectra indicate changes in base stacking and unwinding of the helix conformation of CT-DNA. These types of alterations in the CD signals can be assigned to modifications on the secondary structure of DNA and offer additional evidence that complex 2 binds at the groove of the DNA.

#### 3.5.2. Absorption Titration

**Figure 5:**
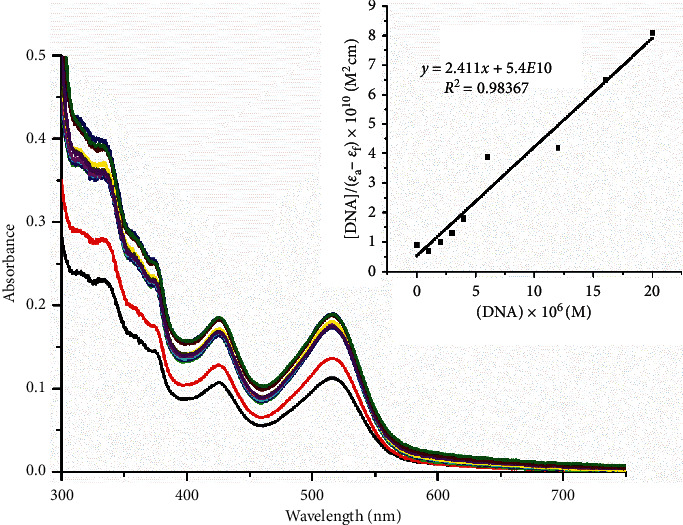
Absorption titration for complex 2 with DNA.

The absorption spectra of ratios in Tris-HCl buffer with constant concentration of the complex (20 *μ*Μ) and increasing concentration of DNA from 0 to 400 *μ*Μ can be found at Figure 5. The absorbance peaks at 488 nm and 514 nm attributed to MLCT transitions of the complex, after the addition of specific amounts of DNA, continuously increased with a hypsochromic shift. Furthermore, the intensity of bands at 345 nm and 380 nm, which are attributed to ILCT transitions [22], shows a similar behavior with the MLCT of complex 2. Hyperchromism with a very slight blue shift can be attributed to the adoption of the appropriate conformation of the complex in which the aromatic system of the rings could connect by bonds that match the torsion of the grooves of DNA. It can be well assumed that such interaction affects the absorption bands of the complex, by the hyperchromism observed at 514 nm in about 50%, which suggests that the complex interacts with DNA by groove binding.

The binding constant *K*_b_ was calculated, from three different experiments, as the ratio of slope of the plot of (DNA)/(*ε*_*a*_−*ε*_*f*_) vs. (DNA) to the intercept at 514 nm and equals to 4.46 × 10^5^ M^−1^ (Table 6). This value shows a great binding affinity for DNA and comes in agreement with other metal complexes that contain the 2, 2′-pq ligand [59]. In addition, this value is greater than the *K*_b_ for [Ru(bpy)_2_(phen)]^2+^, [Ru(bpy)_3_]^2+^, and [Ru(phen)_3_]^2+^, which equals to 9.1·10^3^ M^−1^, 0.248·10^4^ M^−1^, and 0.349·10^4^ M^−1^, respectively [3, 60, 61], indicating that the 2, 2′-pq ligand plays a significant role in the interactions with DNA. On the other hand, the Kb of 2 is ten times lower than the Kb of typical intercalators as [Ru(bpy)_2_ (dppz)]^2+^ (*K*_b_ > 10^6^ M^−1^) [62] and [Ru(phen)_2_(dppz)]^2+^ (*K*_b_ = 6· 10^7^ M^−1^) [63] and further supports a groove binding mode. Furthermore, by applying the equation ΔG = −RTlnK_b_, where *R* is the gas constant, *T* = 298 K, and *K*_b_ is the calculated binding constant, ΔG is calculated at −32.22 KJ/mol^−1^ (−7.7 Kcal/mol), indicating a spontaneous interaction among the complex and the CT-DNA, whereas for EB, the ΔG value is estimated at −8.7 Kcal/mol [64].

#### 3.5.3. Viscosity Measurements

**Figure 6:**
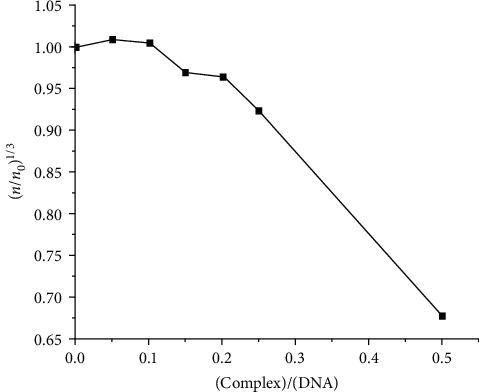
Relative viscosity of DNA versus DNA/complex 2 ratio.

Viscosity measurements were performed to further probe the interaction between 2 and CT-DNA. In order to test the reproducibility of the results, three different measurements were taken for each ratio differing less than 0.3 s.  Figure 6 shows the relative viscosity of DNA versus *r* ratio. A glaring decrease is observed, up to 0.68, as the concentration of complex increases, corroborating previous indications that the complex binds in the grooves of the double helix reducing its effective length, hence its viscosity. Thus, an intercalative DNA binding mode could unequivocally be excluded.

#### 3.5.4. Competitive Studies with Ethidium Bromide

**Figure 7:**
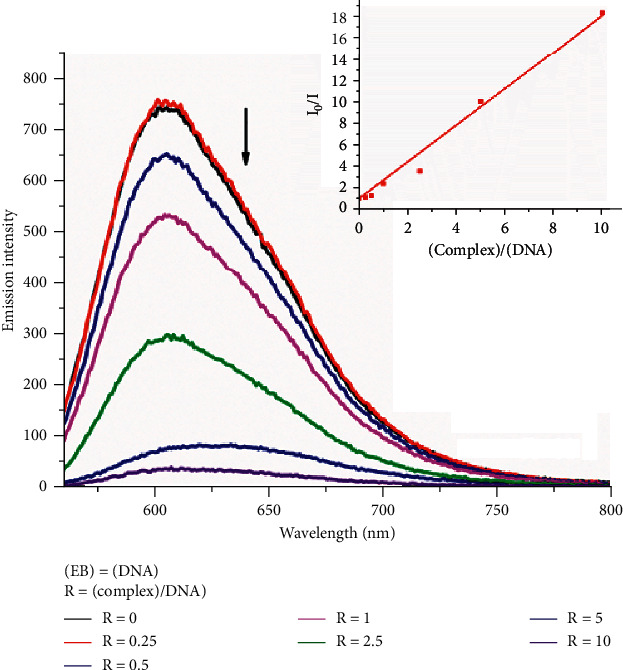
Emission spectra of EB bound to DNA with increasing amount of 2. (EB) = (DNA) = 1·10^−4^ M [Ru(bpy) (phen) (2, 2'-pq)]^2+^ = 0-10·10^−4^ M *λ*_ex_ = 526 nm, in tris-HCl buffer solution. Stern–Vomer plot of *I*_0_/*I* vs. r (where *r* = (complex 2)/(DNA)) of the fluorescence titration.

With an increasing amount of complexes 1 and 2, a decrease in the emission intensity is observed (Figure 7, Figure S3 for 1), which is associated to the competitive ability of the complexes. Also, at high concentrations (*c* > 2·10^−4^ M), complexes 1 and 2 destabilize the DNA-EB complex, leading to a significant decrease in fluorescence emission. The extent of fluorescence quenching of EB-DNA does not reflect the extent of the intercalative mode of ruthenium complexes because at lower or equal concentrations, only inadequate quenching is observed (*r* = (EB)/(complex 2) = 1 at 23% decrease in emission intensity). The quenching of bound EB-DNA can be attributed to potential energy or energy transfer reactions among it and the complexes. The quenching of EB bound to DNA by compounds 1 and 2 is in agreement with linear Stern–Volmer equation [65].

(2) $$\begin{array}{c} I_{0}/I=1+K_{\text{SV}}\left[\text{complex}\right]=1+K_{q}\tau_{\text{o}}\left[Q\right], \end{array}$$

where *I*_o_ and *I* represent the fluorescence intensities in absence and in presence of quencher, respectively; K_sv_ is the Stern–Volmer constant; *K*_*q*_ is the quenching rate constant; and *τ*_*ο*_ is the average lifetime of DNA without the quencher molecule (*τ*_*ο*_ = 10^−8^ s) [65, 66]. From the slope of the regression line in the derived plot (*I*_o_/*I* vs. *r* = (complex)/(DNA)), the K_SV_ value for each complex is calculated at 0.87 M^−1^ and 0.96 M^−1^ for 1 and 2, respectively, indicating that 2 exhibits better affinity to CT-DNA, probably due to the phen moiety's contribution to the mechanism of the interaction. The *K*_sv_ values (Table 6) confirm groove binding and are in the same order of magnitude as other ruthenium complexes with groove binding ability [67, 68].

**Figure 8:**
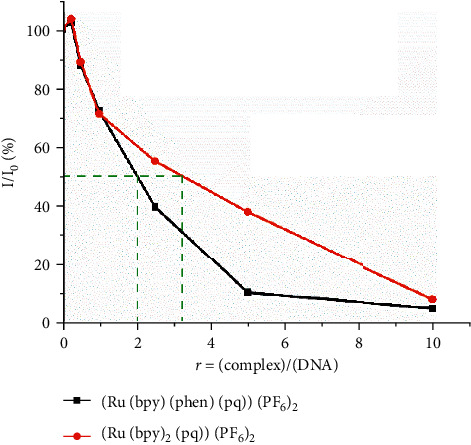
The % relative intensity of fluorescence emission of EB at *λ*_em_ = 605 nm vs. r (*r* = (complex)/(CT-DNA)) for the complexes 1 and 2 in tris-HCL buffer (150 mM NaCl, pH = 7.4).

Moreover, from equation (1), the quenching rate constant, *K*_*q*_ is calculated as 10^11^ M^−1^ and 10^12^ M^−1^ for 1 and 2, respectively, pointing to a static quenching mechanism, since the limitation for the dynamic quenching process is at *K*_*q*_ = 2.0·10^8^ M^−1^. However, further experiments at different temperatures should be conducted to verify the quenching mechanism. On the other hand, the decrease in the intensity of emission is related to the binding affinity of the complexes to DNA and can be used to calculate their binding constant, following the equation [69, 70]:

(3) $$\begin{array}{c} K_{\text{EB}}{\left[\text{EB}\right]}_{50\%}=K_{\text{app}}{\left[Q\right]}_{50\%}, \end{array}$$

where *K*_app_ is the apparent DNA binding constant of 1 or 2, *K*_EB_ is the DNA binding constant of EB (equals to 1.25·10^6^ M^−1^), and [EB]_50%_ and [Ru]_50%_ are the EB and complexes 1/or 2 concentrations at which 50% of EB molecules in EB-DNA were displaced [54]. As shown in Figure 8, the decrease in the emission intensity up to 50% is at *R* = 3 and *R* = 2 for 1 and 2, respectively (*R* = (complex)/(EB-DNA)) indicating that both complexes bind less strong than EB to DNA, therefore not displacing EB, and consequently, a groove binding mode is suggested. These findings come in accordance with the results of the above experiments.

#### 3.5.5. Cytotoxicity Studies

In vitro studies tested the antiproliferative activity of 1 and 2 against MCF-7 cell line (human breast cancer cell line) and healthy (HEK-293, human embryonic kidney healthy cells 293). IC_50_ values of both complexes and cis-platin for both cell lines were calculated, and the results are summarized in Table 7. Between the two complexes, 2 presents the highest activity against MCF-7 cells (IC_50_ = 6.2 ± 1.2 *µ*Μ), almost equal to cis-platin (IC_50_ = 5.19 ± 0.8 *µ*Μ); whereas, both complexes cause almost no apoptosis to HEK-293 (at least, to the tested concentrations). The presence of phen ligand also induces a difference in activity of 2 compared to 1, which can be attributed to the greater hydrophobicity of phen compared to bpy and at the same time highlights the potency of fused heterocyclic moieties in designing metallodrugs [73]. The observed antitumor activity of both complexes is better than other reported bis-1,10-phenanthroline or bipyridyl Ru complexes and comparable with other metallodrugs carrying the 2,2′-pq ligand (Table 7), highlighting the potency of 2,2′-pq moiety in antiproliferative activity.

**Figure 9:**
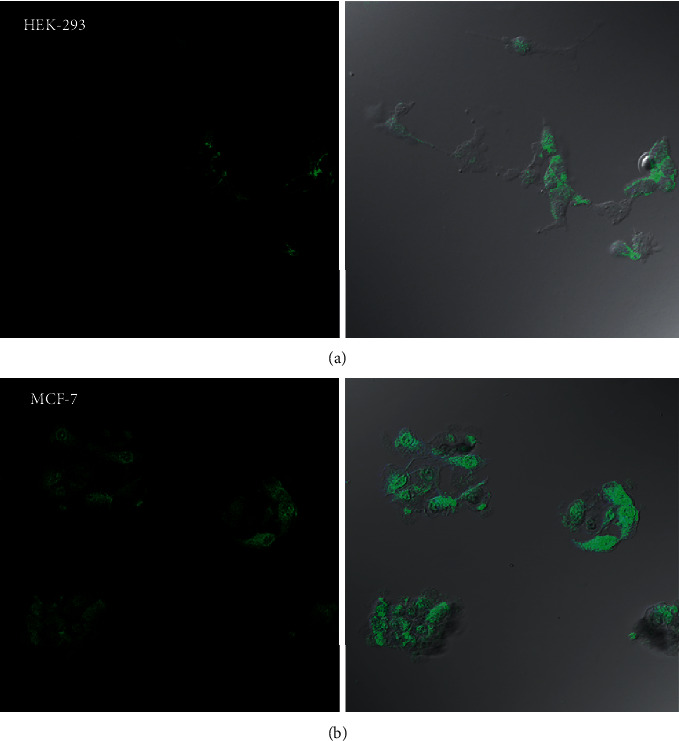
Confocal microscopy images of 2 about cellular uptake and the localization mode in healthy (HEK-293) (a) and breast cancer cells (MCF-7) (b).

Besides, confocal images (Figure 9) indicate that complexes 1 and 2 can localize on both cell lines, and the localization mode depends on the cell line. Thus, in HEK-293, complex 2 is localized on the cytoplasm; whereas, it is localized both on the cytoplasm and nucleus to MCF-7 cells. The latter indicates that 2 internalizes most efficiently in MCF-7 cells and can act as an antitumor agent.

#### 3.5.6. Molecular Docking of the Complex with DNA Sequence

**Figure 10:**
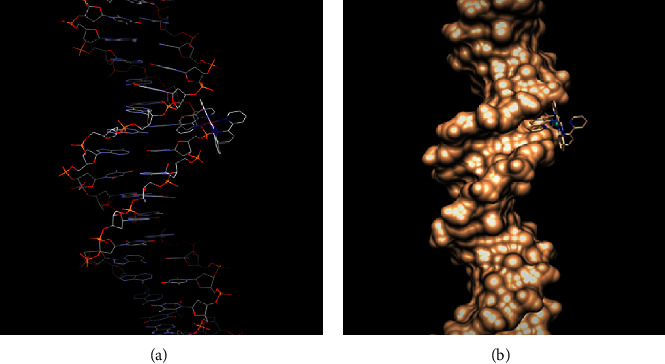
(a) Visualization of the interaction of 2 via phen moiety to the specific region of the sequence of DNA (5D2Q). (b) Interaction of complex 2 through the phen moiety with the hydrophobic DNA sequence (PDB: 5D2Q).

**Figure 11:**
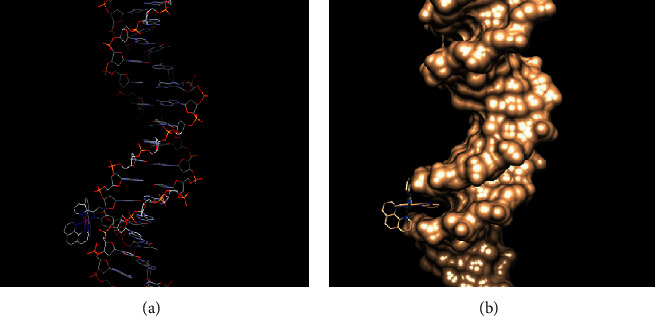
(a) Visualization of the interaction of 2 via 2, 2'-pq moiety to the specific region of the sequence of DNA (5D2Q). (b) Interaction of complex 2 through the 2, 2'-pq moiety with the hydrophobic DNA sequence (PDB: 5D2Q).

Molecular docking is an appealing technique to shed light into drug-biomolecule interactions. Taking into account the experimental data of DNA binding for 1 and 2, molecular docking studies were performed in order to identify the most preferable binding site on the biomolecule, assuming that DNA and ruthenium complexes are rigid. Complex 1 was already studied through 1D and 2D NMR, indicating that both isomers Λ- and Δ-interact with oligonucleotides by the same mode and can provide us a good indicator for our docking calculations [21]. The selected optimized structures of complexes 1 and 2 obtained from DFT calculations in the gas phase were used in docking experiments, utilizing crystallography data. A series of DNA duplex sequences known for groove binding were tested, and the ones with the lower (more stable) energy are given thereafter. The results of docking are presented in Table 8 and confirm the experimental data. According to our docking calculations, both complexes 1 and 2 interact successfully with DNA duplex sequences (TCATAAATGTATCTAAGTAG)_2_ and (ACCGACGTCGGT)_2_ by minor groove binding. Actually, complex 2 docks to the former sequence by two different modes, through the *π*-aromatic system of phen and 2, 2′-pq within the T*-*A (Figure 10) and C-T regions (Figure 11), respectively. Moreover, in nine out of twenty runs, binding is performed through the *π*-aromatic system of 2, 2′-pq, which interacts with the aromatic system of thymine T4 of B-helix. On the other hand, the *π*-aromatic system of phen interacts both with the aromatic systems of the sugar and the thymine T12 of B-helix. The bpy moiety does not intercalate but points across to the opposite strand. These results are in accordance with the fact that regions of A-T bases are suitable for ligands making optimal Van der Waals contacts and with the literature of Ru(II)-diimine complexes [23, 74, 75]. The binding energy is at −7.33 kcal∙mol^−1^ and −7.18 kcal∙mol^−1^when 2 binds through phen and 2, 2'- pq ligand, respectively. These conclusions are in accordance with the calculated ΔG from the data of UV-Vis (vide supra).

**Figure 12:**
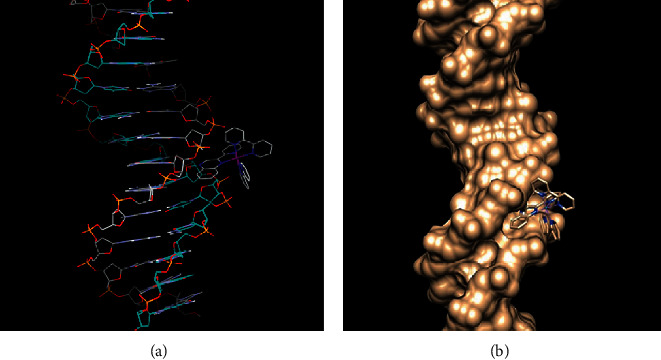
(a) Visualization of the interaction of 1 via 2, 2'-pq moiety to the specific region of the sequence of DNA (PDB: 5D2Q). (b) Interaction of complex 1 through 2, 2'-pq moiety with the hydrophobic DNA sequence (PDB: 5D2Q).

As far as (ACCGACGTCGGT)_2_ sequence is concerned, the binding sites are slightly different, since the *π*-aromatic system of benzene ring of 2,2′-pq moiety of 2 matches with the *π*-aromatic system of cytosine of A-helix of DNA (423D : A : DC9) but not with phen, which stands against the hydrophilic chain (Figure S4). Van der Waals interactions enhance the stabilization of complex with DNA. On the other hand, complex 1 adducts with both sequences of DNA via 2, 2′-pq moiety and the binding energy is −7.03 Kcal·mol^−1^ and −6.76 kcal·mol^−1^, respectively (Table 8). Regarding the (TCATAAATGTATCTAAGTAG)_2_ sequence, the *π*-aromatic system of 2, 2′-pq of 1 interacts with the *π*-aromatic system of thymine of the B-helix of DNA (5D2Q : B:DT16) (Figure 12); whereas, it interacts with the (ACCGACGTCGGT)_2_ sequence through the *π*-aromatic thymine of the A-helix of DNA (423:B : DT20) (Figure S5). Simultaneously, a strong H-bond and average bond length 1.765 Å is formed between the hydrogen atom of guanine (A : DG67 : 21) and the nitrogen of quinoxaline (Figure S6, Table 9). This is in accordance with previous findings for complex 1 [23]. Additionally, NMR studies in ruthenium complexes substituted with 2,2′-pq ligand reveal the minor groove binding mode. The octahedral geometry of ruthenium complexes does not favor the efficient interference of the bulkier 2,2′-pq ligand [76].

## 4. Conclusions

In conclusion, two heteroleptic ruthenium (II) polypyridyl complexes, 1 and 2, were prepared in an efficient, stepwise manner, and their photophysical and electrochemical properties were investigated. The electrochemical data reveal that the trisheteroleptic complex is easily reduced than its bis-analog based on the different electronic properties of the ligands. Complex 2 interacts with cellular DNA via the minor groove binding mode as evidenced by various spectroscopic techniques, hydrodynamic experiments, and molecular docking studies. The interaction of 1 with DNA had been previously reported by NMR spectroscopy but is well documented through this study. The docking experiments reveal that both complexes interact as minor groove binders with the duplex sequences (TCATAAATGTATCTAAGTAG)_2_ and (ACCGACGTCGGT)_2_ with some differences in the binding mode. Complex 2 binds through the 2,2′-pq and phen ligand with the former to prefer the thymine and the sugar bases and the latter cytosine site. The theoretical calculated binding energies are in agreement with the experimental one (−7.7 kcal∙mol^−1^). On the other hand, 1 interacts with both sequences through 2, 2′-pq ligand with a preference to thymine. Finally, in vitro experiments reveal the significant role of the 2,2′-pq in the cytotoxicity of both complexes. Both complexes act as promising antitumor agents with complex 2 to localize in the nucleus and have better activity than 1 and cis-platin. More extensive theoretical studies are underway for a rational ligand design of metallodrugs.

## Figures and Tables

**Table 1:** Crystal data and structure refinement for 1 and 2.

Identification code	1	2
Empirical formula	C_34_H_27_Cl_2_F_12_N_7_O_0.5_P_2_Ru	C_35.7_H_26.4_Cl_1.4_F_12_N_7_O_0.3_P_2_Ru
Formula weight	1003.54	998.88
Temperature	293(2) K	293(2) K
Wavelength	0.71073 A	0.71073 A
Crystal system, space group	Orthorhombic, Pbca	Monoclinic, P2_1_/*n*
Unit cell dimensions	*a* = 28.44(1) A alpha = 90 deg.	*a* = 11.026(1) A alpha = 90 deg.
	*b* = 19.332(9) A beta = 90 deg.	*b* = 14.398(1) A beta = 90.284(1) deg.
	*c* = 14.369(7) A gamma = 90 deg.	*c* = 24.972(2) A gamma = 90 deg.
Volume	7900(6) A^3^	3964.3(6) A^3^
Z, calculated density	8, 1.689 Mg/m^3^	4, 1.674 Mg/m^3^
Absorption coefficient	0.708 mm^−1^	0.666 mm^−1^
F(000)	4008	1991
Crystal size	0.31 × 0.24 × 0.12 mm	0.32 × 0.17 × 0.15 mm
Theta range for data collection	1.78–24.75 deg.	1.63–25.16 deg.
Limiting indices	−33 ≤ *h* ≤ 33, −22 ≤ *k* ≤ 22, −16 ≤ *l* ≤ 16	13 ≤ *h* ≤ 13, −17 ≤ *k* ≤ 17, −29 ≤ *l* ≤ 29
Reflections collected/unique	56369/6722 (R(int) = 0.0820)	42163/7067 (R(int) = 0.0732)
Data/restraints/parameters	6722/5/569	7067/248/664
Goodness-of-fit on F^2^	1.032	1.078
Final *R* indices (I > 2sigma(I))	*R*_1_ = 0.0657, wR_2_ = 0.1604	*R*_1_ = 0.0655, wR_2_ = 0.1455
Largest diff. peak and hole	2.22 and −1.01 e.A^−3^	0.59 and −0.53 e.A^−3^

**Table 2:** Spectroscopic properties for complexes 1 and 2.

Compound	*λ*_max._ (nm)^a^	*ε* (Μ^−1^ cm^−1^)	*λ*em (nm)^b^
1	508	10800	755
	429	9730	
2	507	10100	755
	428	9700	

^a^Measured in MeCN in a 1.0 cm cuvette. ^b^Measured in MeCN in a 1.0 cm cuvette, excitation at 500 nm.

**Table 3:** *E*_1/2_ values for complexes 1 and 2.

Compound	*E*_1/2_^a^			
1	1.190	−1.023	−1.678	−1.890
2	1.343	−0.889	−1.529	−1.743

^a^Values in volts, measured in an MeCN solution with a glassy carbon electrode, an Ag wire pseudoreference electrode vs. the Fc^+/0^ redox couple with NBu_4_PF_6_ supporting electrolyte, 100 mV/s scan rate.

**Table 4:** Selected Ru-N bond lengths (Å).

	1	2
Ru-N11	2.063(5)	2.048(5)
Ru-N21	2.119(5)	2.098(5)
Ru-N23	2.073(5)	2.069(5)
Ru-N13	2.061(5)	2.065(5)
Ru-N22	2.073(5)	2.062(5)
Ru-N12	2.075(5)	2.060(5)

**Table 5:** Metal-N_quinoxaline_ and metal-N_pyridyl_ bond lengths (Å) for 2, 2'-pq complexes.

Compound	Metal-N_quinoxaline_	Metal-N_pyridyl_	Ref.
[W(CO)_5_(2, 2'-pq)]	2.299	2.202	[50]
[Mo(CO)_5_(2, 2'-pq)]	2.300	2.220	[46]
[Re(CO)_5_(Cl-2, 2'-pq)Cl]	2.230	2.175	[47]
[Re(CO)_5_(2, 2'-pq)Br]	2.220	2.145	[48]
[Pt(2, 2'-pq)Cl_2_]	2.079	2.029	[51].
[Ag(2, 2'-pq)_2_]ClO_4_	2.260	2.407	[52]
[Ru(bpy)_2_(2, 2'-pq)](PF_6_)_2_ (1)	2.119	2.063	This study
[Ru(bpy) (phen) (2, 2'-pq)](PF_6_)_2_ (2)	2.097	2.049	

**Table 6:** Binding constants and Stern–Volmer quenching constants of Ru (II) complexes at 25°C.

Compound	*K*_b_ (M^−1^)	K_SV_ (M^−1^)	*K*_q_ (M^−1^s^−1^)	K_app_ (M^−1^)	Βinding mode	References
1		0.87	3.4·10^11^	6·10^4^		
2	4.46·10^5^	0.96	1.8·10^12^	9·10^4^		
[Ru(phen)_2_(cd2, 2'-pq)]^+2^	4.67 10^3^	0.052		4.67·10^3^	Groove binding	[56]
[RuCl_3_(dmso) (phen)]		3.03		6.9·10^6^	Intercalation	[57]
[RuCl_3_(dmso) (bpy)]		1.73		3.43·10^6^		[57]
[RuCl_3_(dmso) (dppz)]		4.47		8.62·10^6^	Intercalation	[57]
[Ru(aeip)_2_(Haip)]^2+^	4.47·10^6^	3.26			Groove binding	[58]

**Table 7:** In vitro activity of 1, 2, and cis-platin (expressed as IC_50_ (*μ*M) against MCF-7 cells).

Complexes	MCF-7	HEK-293	References
1^*∗*^	10.5 ± 0.9	n.a	
2^*∗*^	6.2 ± 1.2	n.a	
Cis-platin	5.19 ± 0.8	6.53 ± 1.1	
[Ru(bip)_2_Cl_2_]	134.9 ± 7.9		[71]
[Ru(bmp)_2_Cl_2_]	>200		[71]
[RuCl_3_(dmso) (bpy)]	691		[57]
[RuCl_3_(dmso) (phen)]	679		[57]
cis-[Ru(bpy)_2_Cl_2_]	189.2		[72]
cis-[Ru(phen)_2_Cl_2_]	>200		[72]
[Cu(pq) (NO_3_)]NO_3_	17.4 ± 1.1		[59]
[Cu(pq)_2_(NO_3_)]NO_3_·6H_2_O	4.92±		[59]

^*∗*^The cytotoxicity study is performed on amorphous material. The IC50 values are the average of three separate experiments_._ n.a., not active (IC_50_ > 100 *μ*M);

**Table 8:** Molecular docking studies for 1 and 2 with the sequence (TCATAAATGTATCTAAGTAG)_2_ (pdb code: 5D2Q) and (ACCGACGTCGGT)_2_ (pdb code: 423D).

Pdb code	Complexes	Ligand moiety	Binding energy (kcal·mol^−1^)	Intermolar energy (kcal·mol^−1^)	Electrostatic energy (kcal·mol^−1^)	Inhibition constant (*μ*M)
5D2Q	(1)	2, 2'-pq	−7.03	−7.03	−0.04	7.05
	(2)	phen	−7.33	−7.33	−0.04	4.22
		2, 2'-pq	−7.18	−7.18	−0.08	5.49
423D	(1)	2, 2'-pq	−6.76	−6.76	−0.08	11.02
	(2)	phen	—	—	—	—
		2, 2'-pq	−6.19	−6.19	−0.07	29.22

**Table 9:** Molecular docking studies for 1 and 2 with the sequence (ACCGACGTCGGT)_2_ (pdb code: 423D).

Atoms that participate	Distances	Energy	
A : DG67 : 21 and N of quinoxaline	1.783	7.683	1
	—	—	2

## Data Availability

Crystallographic data for the structures reported in this study are deposited with the Cambridge Crystallographic Data Centre under the CCDC numbers: 2052212 ([Ru(bpy)2(2, 2′-pq)](PF6)2 (1)) and 2052213 ([Ru(bpy) (phen) (2, 2′-pq)](PF6)2 (2)). Copies of these data can be obtained free of charge from http://www.ccdc.cam.ac.uk/data_request/cif.

## Abbreviations

aeip: 2-(Anthracen-9-yl)-1-ethyl-imidazo[4,5-f][1, 10]phenanthroline
^1^H NMR: Proton nuclear magnetic resonance spectroscopy
bpy: 2,2′-Bipyridine phen = 1,10-phenanthroline
2: 2′-pq = 2-(2′-Pyridyl)-quinoxaline
CD: Circular dichroism
CT-DNA: Calf thymus DNA
dip: 4,7-Diphenyl-1,10-phenanthroline
dmp: 2,9-Dimethyl-1,10-phenanthroline
DMF: *N,N*-Dimethylformamide
DMSO: Dimethylsulfoxide
EB: Ethidium bromide
Haip: 2-(9-Anthryl)-1H-imidazo[4,5-f][1,10]phenanthroline
HEK-293: Human embryonic kidney healthy cells 293
HRMS: High resolution mass spectroscopy
ILCT: Interligand charge transfer
K_SV_: Stern–Volmer constant
MCF-7: Cell line (human breast cancer cell line
MLCT: Metal-to-ligand charge transfer
MTT: Methyl thiazolyl tetrazolium
PAF: Platelet activating factor
TGA: Thermogravimetric analysis.

## Supplementary Materials

Supplementary Materials Crystallographic data for the structures reported in this study are deposited with the Cambridge Crystallographic Data Centre under the CCDC numbers: 2052212 ([Ru(bpy)2(2, 2′-pq)](PF6)2 (1)) and 2052213 ([Ru(bpy) (phen) (2, 2′-pq)](PF6)2 (2)). Copies of these data can be obtained free of charge from http://www.ccdc.cam.ac.uk/data_request/cif.
