# Supplementary material for: Synthesis, DNA-Binding, Anticancer Evaluation, and Molecular Docking Studies of Bishomoleptic and Trisheteroleptic Ru-Diimine Complexes Bearing 2-(2-Pyridyl)-quinoxaline
Source: Bioinorg Chem Appl. 2021 May 12;2021:5599773. doi: 10.1155/2021/5599773 (PMC8137304; doi:10.1155/2021/5599773)
Supplement: Supplementary Materials — Crystallographic data for the structures reported in this study are deposited with the Cambridge Crystallographic Data Centre under the CCDC numbers: 2052212 ([Ru(bpy)2(2, 2′-pq)](PF6)2 (1)) and 2052213 ([Ru(bpy) (phen) (2, 2′-pq)](PF6)2 (2)). Copies of these data can be obtained free of charge from http://www.ccdc.cam.ac.uk/data_request/cif. [file 5599773.f1.DOCX]

Supporting Information for

**DNA-binding, anticancer evaluation, and molecular docking studies of bis- and tris-heteroleptic Ru-diimine complexes bearing 2-(2'-pyridyl)-quinoxaline**

Sofia Balou^a,#^, Athanasios Zarkadoulas^a,#^, , Maria Koukouvitaki^a^, Luciano Marchiò^b^, Eleni K. Efthimiadou^a,c^, Christiana A. Mitsopoulou^a^*

*^a^ Inorganic Chemistry Laboratory, Chemistry Department, National and Kapodistrian University of Athens, Panepistimiopolis, Zografou 157 71, Greece*

*^b^Dipartimento di Scienze Chimiche, della Vita e della Sostenibilità Ambientale, Università degli Studi Parma, Parco Area delle Scienze 17A, I43124 Parma, Italy*

*^c^* Institute of Nanoscience and Nanotechnology, [National Center for Scientific Research Demokritos](https://www.researchgate.net/institution/National_Center_for_Scientific_Research_Demokritos)

*^#^: these authors contributed equally*

**Table of Contents**

[Experimental Methods 1](#_Toc67653595)

[1. Synthesis of the Ru precursors 1](#_Toc67653596)

[1.1 Synthesis of *cis*-[Ru(bpy)(DMSO)_2_Cl_2_] 1](#_Toc67653597)

[1.2. Synthesis of *cis*-[Ru(bpy)(phen)Cl_2_] 1](#_Toc67653598)

[1.3 Thermogravimetric Analysis (TGA). 1](#_Toc67653599)

[Figure S1. Thermogravimetric analysis curves of complexes 1 (top) and 2 (bottom) 2](#_Toc67653600)

[Table S1. Thermogravimetric data for decomposition of 1 and 2 complexes. 3](#_Toc67653601)

[Figure S2. HRMS spectra of 1 and 2 with the compass IsotopePattern windows of complexes indicating the theoretical mass spectrum(m/z) and the isotope profiles of both complexes. 3](#_Toc67653602)

[2. DNA binding Studies 4](#_Toc67653603)

[2.1 Circular dichroism (CD) measurements. 4](#_Toc67653604)

[2.2 Absorption titration. 4](#_Toc67653605)

[2.3. Viscosity measurements. 5](#_Toc67653606)

[2.4 Fluorescence emission spectroscopy. 5](#_Toc67653607)

[3. In vitro Cytotoxicity Studies. 5](#_Toc67653608)

[3.1 MTT assay 5](#_Toc67653609)

[3.2 Confocal microscopy. 6](#_Toc67653610)

[3.2. Molecular docking study. 6](#_Toc67653611)

[Figure S3. Emission spectra of EB bound to DNA with increasing amount of 1. [EB] = [DNA] = 1·10^-4^ M, [Ru(bpy)_2_(pq)]^2+^ = 0-10·10^4^ M, λex = 526 nm, in tris – HCl buffer solution. Stern-Vomer plot of I_0_/I vs r, (r = [complex 1] / [DNA]) of the fluorescence titration. 7](#_Toc67653612)

[Figure S4. A: visualization of the interaction of 1 via pq moiety to specific region of the DNA sequence II, B: interaction of complex 1 through the pq moiety with the hydrophobic sequence II. 8](#_Toc67653613)

[Figure S5. A: visualization of the interaction of 2 via pq moiety to specific region of the sequence II, B: interaction of complex 2 through pq moiety with the hydrophobic DNA sequence II. 8](#_Toc67653614)

[Figure S6. Visualization of the formation of H-bond of 1, through nitrogen atom of quinoxaline and hydrogen atom of guanine 7 of sequence ΙΙ. 9](#_Toc67653615)

[References 9](#_Toc67653616)

# **Experimental Methods**

# **1. Synthesis of the Ru precursors**

# **1.1 Synthesis of *cis*-[Ru(bpy)(DMSO)_2_Cl_2_]**

149 mg (0.3075 mmol) of *cis*-[Ru(DMSO)_4_Cl_2_] were suspended in 15 mL toluene and to this suspension was added a solution of bpy (48 mg, 1 eq.) in 5 mL toluene. The mixture was then refluxed under stirring for 2 h, cooled to room temperature, and filtered in a sintered funnel. After washing with ether and air-drying, 125.2 mg of the desired product as a brown-yellow solid were isolated. Yield: 84%. Anal. Found (Calc.) for C_14_H_20_Cl_2_N_2_O_2_RuS_2_: C, 34.89 (34.71); H, 4.21 (4.16); N, 5.81 (5.78); ^1^H-NMR (CDCl_3_, ppm): 9.90 (d, 1H, bpy), 9.70 (d, 1H, bpy), 8.04-8.11 (m, 3H, bpy), 7.93 (t, 1H, bpy), 7,60 (t, 1H, bpy), 7,47 (t, 1H, bpy), 3.55, (s, 3H, DMSO), 3.52 (s, 3H, DMSO), 3.23 (s, 3H, DMSO), 2.69 (s, 3H, DMSO).

# **1.2. Synthesis of *cis*-[Ru(bpy)(phen)Cl_2_]**

100 mg (0.206 mmol) of *cis*-[Ru(bpy)(DMSO)_2_Cl_2_], 37.2 mg (1 eq.) of phen, and 87.5 mg (10 eq.) LiCl were dissolved in 8 mL of N_2_-purged DMF. The solution was refluxed for 4 hours, and after removal of the solvent, 20 mL of methanol were added to the residue. The solution turned red and stirred for 30 min at room temperature; then a mixture of 30 mL ether – 10 mL hexane was added. After staying overnight at -40 ^o^C, the mixture was filtered in a sintered funnel to afford 47 mg of the desired compound as a purple solid. Yield: 47%. Anal. Found (Calc.) for C_22_H_16_Cl_2_N_4_Ru: C, 51.36 (51.98); H, 3.06 (3.17); N, (11.02). ^1^H-NMR (DMSO-d_6_, ppm): 10.18 (d, 1H), 10.07 (d, 1H), 8.67 (d, 2H), 8.48 (d, 1H), 8.29-8.12 (m, 5H), 7.87-7.81 (m, 2H), 7.63 (t, 1H), 7.47 (t, 1H), 7.57 (d, 1H), 6.96 (t, 1H).

# **1.3 Thermogravimetric Analysis (TGA).**

The thermal analysis was conducted with approximately 3.4 mg of each sample in an alumina crucible, with a nitrogen flow of 70 mL min^−1^ and a heating ramp of 10 °C min^−1^ in a temperature range of 25–800 °C. The results of the temperature ranges of decomposition along with the corresponding mass losses of species are given in Table S1. Both complexes exhibit high thermal stability which is reflected by the relatively great TGA data which are presented in Fig. S1. The thermogram of **1** shows two main mass loss steps, whereas no loss of solvent or water molecules is observed. The first step, which occurs between 250 and 420 °C, shows a weight loss of approximately 31.9 %, which is attributed to the loss of two PF_6_^-^ ions. The second step which occurred between 420 and 800 °C, corresponds at an approximately 48.2 % weight loss, which is attributed to the loss of the quinoxaline ring of pq with the two bpy ligands. Thermogravimetric analysis (TGA) of **complex 2** revealed that thermal decomposition occurs in four steps. The first and the second steps are attributed to water loss. In the third step, which occurs between 300 and 400 °C a weight loss of approximately 15.51 % is observed, which is assigned to the loss of the bpy ligand. The final step occurrs between 500-800 °C, which corresponds to a 48.2 % weight loss, and is attributed to the loss of two PF_6_^-^counter ions, phen ligand, and quinoxaline ring from pq. Such thermal stability indicates that both complexes can undergo thermal treatments and remain stable till 100 °C undoubtedly.

**
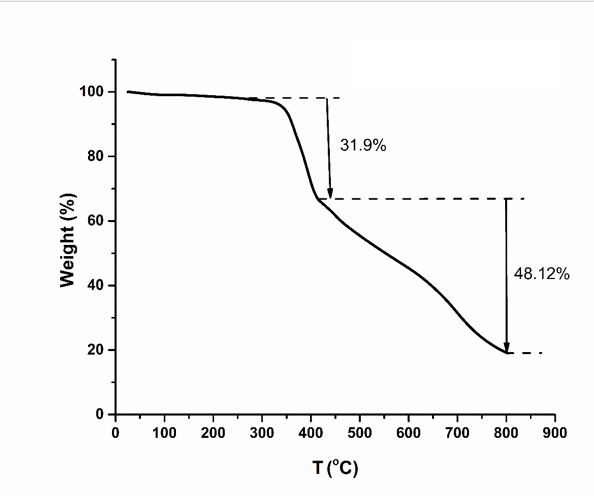

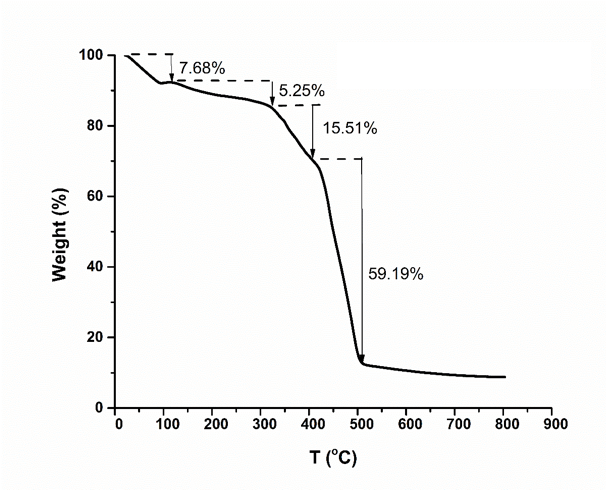
**

**Figure S1. Thermogravimetric analysis curves of complexes 1 (top) and 2 (bottom).**

# **Table S1. Thermogravimetric data for decomposition of 1 and 2 complexes.**

| Complexes | MW | Weight loss % | Mass loss | | Eliminated  Species |
| --- | --- | --- | --- | --- | --- |
|  |  |  | Theoretical | Found |  |
| 1 | 910.41 | 31.90 | 289.93 | 290.48 | 2 PF_6_^-1^ |
|  |  | 48.12 | 441.18 | 438.18 | 2 bpy, quinoxaline ring from pq |
| 2 | 1012.4 | 7.68 | 72.06 | 77.74 | 4 H_2_O |
|  |  | 5.25 | 54.05 | 53.15 | 3 H_2_O |
|  |  | 15.51 | 156.19 | 156.99 | bpy |
|  |  | 59.92 | 616.33 | 599.24 | 2PF_6_^-1^, phen, quinoxaline from pq |

# **Figure S2. HRMS spectra of 1 and 2 with the compass IsotopePattern windows of complexes indicating the theoretical mass spectrum(m/z) and the isotope profiles of both complexes.**

# **2. DNA binding Studies**

All experiments involving DNA interactions with complex **2** were carried out in Tris-HCl buffer pH=7.0 which was prepared by dissolving 0.394 g (2.5 mmol) of Tris(hydroxymethyl)aminomethane hydrochloride (Tris-HCl) and 1.461 g (25 mmol) of NaCl in 500 mL of milli-Q water. Drops of a 1 M NaOH solution were added to adjust pH to 7.0. The stability of **2** in tris-HCl buffer was tested by monitoring UV-Vis spectra for 72 h; the identification of no changes reveals its stability in the assay buffer solutions. CT-DNA stock solution was prepared by dissolving CT-DNA in 5 mM tris-HCl buffer, and its concentration was determined by UV spectroscopy using its molar coefficient ε_258_ = 6600 M^−1^ cm^−1^ at 260 nm. It is indicated that the DNA was free of protein as the ratio at 260:280nm is 1.9:1 [1]. Stock solution, stored at 4°C, was used for no more than 4 days since its preparation; whereas complex **2** was incubated with DNA in all the experiments at 25°C for 24 h.

# **2.1 Circular dichroism (CD) measurements.**

CD measurements were conducted using a Jasco J815 spectropolarimeter interfaced and the Jasco software package was used for analyzing data. Ratios of increasing concentration of the complex from 0 μM to 25 μΜ were prepared, incubated with constant concentration of DNA (50 mΜ) in all ratios. The CD spectrum was monitored in the range between 200 nm and 400 nm with data interval 0.2 nm at 25^o^C. Each sample’s spectra are the average of two independent scans.

# **2.2 Absorption titration.**

Absorption titration experiments were carried out by measuring the absorption of samples with constant concentration of complex (20 μΜ) in Tris-HCl buffer solution containing less than 5% MeOH and increasing ratios of DNA. The concentration of DNA in samples varied from 0 to 400 μΜ. The data extracted from the titration, based upon the variation in absorbance, was used to calculate the intrinsic binding constant (K_b_) of the drug with DNA, in the following expression [2,3]:

[DNA]/ (E_a_-E_f_) = [DNA]/ (E_a_-E_f_) + 1/K_b_(E_b_-E_f_). [DNA] stands for the concentration of DNA, ε_a_, and ε_f_ for the coefficient of the absorption of **2** in the presence and in absence of DNA, respectively, for the concentration value mentioned above, while ε_b_ stands for the coefficient of **2** bound fully to DNA. The binding constant (K_b_) can be determined from the ratio to the slope
1/[ε_a_ − ε_f_] of the plot of [DNA]/E_a_ -E_f_, vs [DNA] which has a Y-axis intercept that equals to
1/Kb[ε_a_ − ε_f_] [4]. The linear least-squares analysis needed was done using Origin Lab 9.0 [5]

# **2.3. Viscosity measurements.**

Viscosity studies were carried out using Schott Gerate AVS 310 Automated Viscometer operated at 25.0 ± 0.1 °C. Samples were prepared in solution buffer, maintaining the concentration of DNA constant at 50 μM and increasing the concentration of the complex from 2.5 μM to 25 μΜ. The flow time was measured using a digital stopwatch and each sample was measured three times. Results differ no more than 0.3 s and their average value was calculated as the recorded flow time. Relative viscosities of DNA either in absence or presence of **2** were calculated according to literature, fitting the equation η/η_0_ = (t − t_0_)/t_DNA_−t_0_), where t_0_ stands for the flow time of the buffer solution free of DNA and t_DNA_ for the flow time of buffer solution in the presence of DNA. Accordingly, t is the flow rate of each sample studied with increasing concentration of **2**. The length of the molecule, L, is calculated by the expression L/L_0_=(η/η_0_)^1/3^ through the relative viscosity when L_0_ stands for the length of the molecule in absence of **2** [4]. Data is demonstrated as (η/η_0_)^1/3^ versus ratio [complex]/[DNA], r.

# **2.4 Fluorescence emission spectroscopy.**

In order to examine the binding mode of **1** and **2**, the EB antagonist assay performed [6,7]. Ethidium bromide (EB), a typical fluorophore, shows a significant increase of fluorescence in presence of DNA due to its intercalation between the base pairs of the double helix [2]. When another molecule is present – apart from EB – that could compete for the same sites in the nucleic acid, a quenching of the emission intensity of EB-DNA is observed [2]. Accordingly, samples of increasing concentration of complex (0 μΜ– 100 μM) in solution buffer in the presence of 10 μM CT-DNA incubated with 10 μΜ EB were prepared for measuring the ability of **2** to quench the fluorescence of EB-DNA. The emission spectra were recorded over the range 540 – 700 nm (λ_ex_ = 524 nm) using an RF-5301PC SHIMADZU spectrometer.

# **3. In vitro Cytotoxicity Studies.**

# **3.1 MTT assay**

The MTT assay was used for testing the growth inhibition of MCF-7, and HEK-293 cells for **1**, **2** and *cis*-platin [8]. For this, both cell lines were seeded in 96-well plates (1.5 x 10^4^ cells in 100 μL of medium/well) in triplicate. After remained to attach overnight, they treated with all complexes for 24 h. Consequently, each plate was incubated for another 4 h at 37 °C, after the medium was replaced with 100 μL of 1 mg/mL 3-(4,5-dimethylthiazol-2-yl)-2,5-diphenyl tetrazolium bromide (MTT) solution. The participated dark-blue formazan crystals were dissolved in 100 μL of DMSO. Cells were incubated in various concentrations of compounds **1, 2** and cis platin from 0.1 to 100 μM. Three independent experiments that had each been repeated two times were used for obtaining data and results are expressed as the percentage (%) of treated cells versus untreated cells.

# **3.2 Confocal microscopy.**

Confocal laser microscopy was used for studying the cellular uptake of each compound. For this, a six-well plate containing DMEM was used to insert each cell line (5∙10^6^ cells/well) after it was grown on 0.22 cm^2^ coverslips. The incubation of cells exposed to compounds **1**, **2** or *cis*-platin, in 10 μM concentration [9,10] was for 1 h at 37 °C. Following, the cells were washed three times with PBS and prepared for confocal imaging by using mountain to avoid photobleaching. For the excitation a Leica TCS SP8 MP, inverted confocal microscope with Acousto-Optical Beam Splitter was used carrying a multiband spectral detector Argon. Excitation took plase @ [458, 476, 488, 496](callto:458,%20476,%20488,%20496) & 514 DPSS 561 nm for red. For excitation @ 780nm Multiphoton IR laser MaiTai DeepSee was employed.

# **3.2. Molecular docking study.**

Docking studies were carried out using MGL tools 1.5.4 with AutoGrid4 and AutoDock4 [11–13] to perform blind docking calculations between both ruthenium (II) complexes and DNA sequence. The coordinates of **1** and **2** were taken from DFT-optimized structures [14] utilizing crystallography data. Both the hexafluorophosphate counter-ion and solvent molecules were omitted for better visualization and only the cationic complexes were taken into account. The crystal structure of B-DNA was obtained from Protein Data Bank (PDB: 5D2Q) and (PDB: 423D). Receptor (DNA) and ligand files were prepared using AutoDock Tools [15,16]. Foremost, water molecules and heteroatoms were erased while Gasteiger charges and polar hydrogen atoms were added to the receptor molecule. The docking process takes into account all possible conformations and all the other parameters were set to default for the docking. According to the Autodock scoring function, the lowest energy docked conformation was chosen as the binding mode. The DNA was closed in a box with a number of grid points in x × y × z 92/82/126 for the PDB code: 5D2Q and 123/80/96 for the PDB code: 423D and a grid spacing of 0.375Å. A Lamarckian genetic algorithm, as implement in AutoDock, was selected to perform docking calculations. Visualization of dock poses was conducted with AutoDock graphics and Chimera molecular graphic programs [12,17].


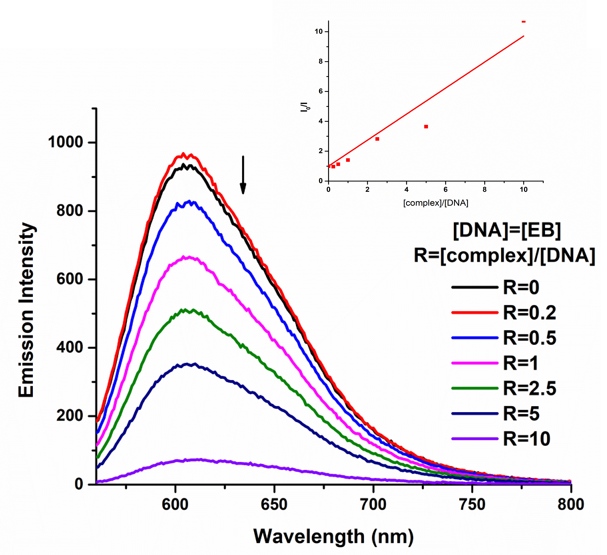


# Figure S3. Emission spectra of EB bound to DNA with increasing amount of 1. [EB] = [DNA] = 1·10^-4^ M, [Ru(bpy)_2_(pq)]^2+^ = 0-10·10^4^ M, λex = 526 nm, in tris – HCl buffer solution. Stern-Vomer plot of I_0_/I vs r, (r = [complex 1] / [DNA]) of the fluorescence titration.


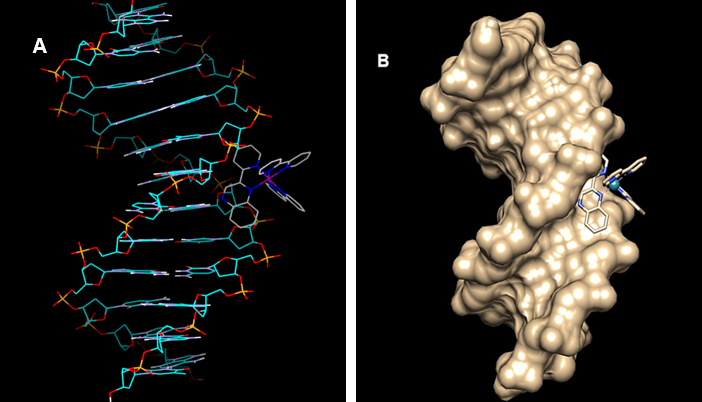


# Figure S4. A: visualization of the interaction of 1 via pq moiety to specific region of the DNA sequence II, B: interaction of complex 1 through the pq moiety with the hydrophobic sequence II.


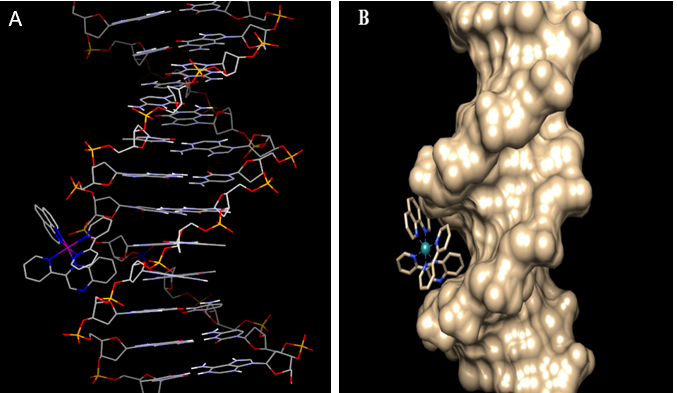


# **Figure S5. A: visualization of the interaction of 2 via pq moiety to specific region of the sequence II, B: interaction of complex 2 through pq moiety with the hydrophobic DNA sequence II.**


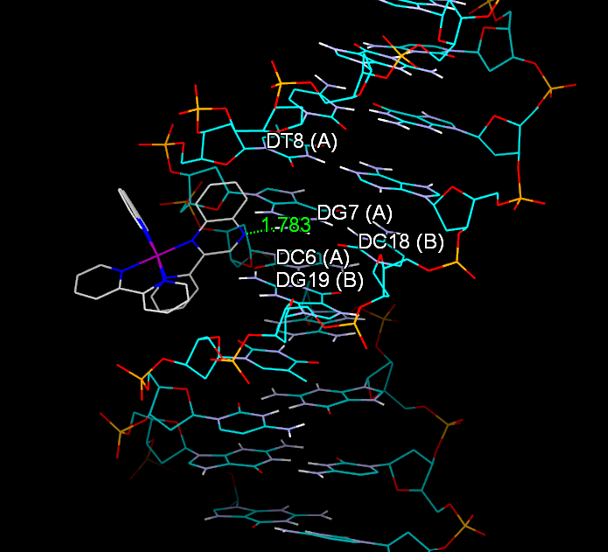


# **Figure S6. Visualization of the formation of H-bond of 1, through nitrogen atom of quinoxaline and hydrogen atom of guanine 7 of sequence ΙΙ.**

# **References**

[1] M.E. Reichmann, S.A. Rice, C.A. Thomas, P. Doty, A Further Examination of the Molecular Weight and Size of Desoxypentose Nucleic Acid, J. Am. Chem. Soc. 76 (1954) 3047–3053. https://doi.org/10.1021/ja01640a067.

[2] M. Sirajuddin, S. Ali, A. Badshah, Drug–DNA interactions and their study by UV–Visible, fluorescence spectroscopies and cyclic voltametry, Journal of Photochemistry and Photobiology B: Biology. 124 (2013) 1–19. https://doi.org/10.1016/j.jphotobiol.2013.03.013.

[3] G. Dougherty, W.J. Pigram, Spectroscopic Analysis of Drug-Nucleic Acid Interaction, Null. 12 (1982) 103–132. https://doi.org/10.3109/10409238209108704.

[4] M. Kaplanis, G. Stamatakis, V.D. Papakonstantinou, M. Paravatou-Petsotas, C.A. Demopoulos, C.A. Mitsopoulou, Re(I) tricarbonyl complex of 1,10-phenanthroline-5,6-dione: DNA binding, cytotoxicity, anti-inflammatory and anti-coagulant effects towards platelet activating factor, Journal of Inorganic Biochemistry. 135 (2014) 1–9. https://doi.org/10.1016/j.jinorgbio.2014.02.003.

[5] J.D. McGhee, P.H. von Hippel, Theoretical aspects of DNA-protein interactions: Co-operative and non-co-operative binding of large ligands to a one-dimensional homogeneous lattice, Journal of Molecular Biology. 86 (1974) 469–489. https://doi.org/10.1016/0022-2836(74)90031-X.

[6] C.A. Mitsopoulou, C. Dagas, Synthesis, characterization, DNA binding, and photocleavage activity of oxorhenium (V) complexes with-diimine and quinoxaline ligands, Bioinorganic Chemistry and Applications. 2010 (2010).

[7] M. Jamshidi, R. Yousefi, S.M. Nabavizadeh, M. Rashidi, M.G. Haghighi, A. Niazi, A.-A. Moosavi-Movahedi, Anticancer activity and DNA-binding properties of novel cationic Pt(II) complexes, International Journal of Biological Macromolecules. 66 (2014) 86–96. https://doi.org/10.1016/j.ijbiomac.2014.01.057.

[8] E.K. Efthimiadou, C. Tapeinos, A. Chatzipavlidis, N. Boukos, E. Fragogeorgi, L. Palamaris, G. Loudos, G. Kordas, Dynamic in vivo imaging of dual-triggered microspheres for sustained release applications: Synthesis, characterization and cytotoxicity study, International Journal of Pharmaceutics. 461 (2014) 54–63. https://doi.org/10.1016/j.ijpharm.2013.11.037.

[9] N.V. Nukolova, H.S. Oberoi, S.M. Cohen, A.V. Kabanov, T.K. Bronich, Folate-decorated nanogels for targeted therapy of ovarian cancer, Biomaterials. 32 (2011) 5417–5426. https://doi.org/10.1016/j.biomaterials.2011.04.006.

[10] A.-F. Metaxa, E.K. Efthimiadou, N. Boukos, E.A. Fragogeorgi, G. Loudos, G. Kordas, Hollow microspheres based on – Folic acid modified – Hydroxypropyl Cellulose and synthetic multi-responsive bio-copolymer for targeted cancer therapy: Controlled release of daunorubicin, in vitro and in vivo studies, Journal of Colloid and Interface Science. 435 (2014) 171–181. https://doi.org/10.1016/j.jcis.2014.08.001.

[11] G.M. Morris, D.S. Goodsell, R.S. Halliday, R. Huey, W.E. Hart, R.K. Belew, A.J. Olson, Automated docking using a Lamarckian genetic algorithm and an empirical binding free energy function, Journal of Computational Chemistry. 19 (1998) 1639–1662. https://doi.org/10.1002/(SICI)1096-987X(19981115)19:14<1639::AID-JCC10>3.0.CO;2-B.

[12] G.M. Morris, R. Huey, W. Lindstrom, M.F. Sanner, R.K. Belew, D.S. Goodsell, A.J. Olson, AutoDock4 and AutoDockTools4: Automated docking with selective receptor flexibility, Journal of Computational Chemistry. 30 (2009) 2785–2791. https://doi.org/10.1002/jcc.21256.

[13] R.H.M. Silva, N. de F.M. Lima, A.J.O. Lopes, C.C. Vasconcelos, J.W.C. de Mesquita, L.S.S. de Mesquita, F.C.V.M. Lima, M.N. de S. Ribeiro, R.M. Ramos, M. do S. de S. Cartágenes, J.B.S. Garcia, Antinociceptive Activity of Borreria verticillata: In vivo and In silico Studies, Frontiers in Pharmacology. 8 (2017) 283. https://doi.org/10.3389/fphar.2017.00283.

[14] M.G. Medvedev, I.S. Bushmarinov, J. Sun, J.P. Perdew, K.A. Lyssenko, Density functional theory is straying from the path toward the exact functional, Science. 355 (2017) 49. https://doi.org/10.1126/science.aah5975.

[15] R. Gaur, R.A. Khan, S. Tabassum, P. Shah, M.I. Siddiqi, L. Mishra, Interaction of a ruthenium(II)–chalcone complex with double stranded DNA: Spectroscopic, molecular docking and nuclease properties, J. Photoch. Photob. A. 220 (2011) 145–152. https://doi.org/10.1016/j.jphotochem.2011.04.005.

[16] R. Rohs, I. Bloch, H. Sklenar, Z. Shakked, Molecular flexibility in ab initio drug docking to DNA: binding-site and binding-mode transitions in all-atom Monte Carlo simulations, Nucleic Acids Research. 33 (2005) 7048–7057. https://doi.org/10.1093/nar/gki1008.

[17] E.F. Pettersen, T.D. Goddard, C.C. Huang, G.S. Couch, D.M. Greenblatt, E.C. Meng, T.E. Ferrin, UCSF Chimera—A visualization system for exploratory research and analysis, Journal of Computational Chemistry. 25 (2004) 1605–1612. https://doi.org/10.1002/jcc.20084.
